# Histoanatomical study of the lens and ciliary body in ostrich eye

**Published:** 2016-09-15

**Authors:** Mohammad Ali Ebrahimi Saadatlou, Rasoul Shahrouz

**Affiliations:** 1 *Department of Basic Sciences, College of Veterinary Medicine, Tabriz Branch, Islamic Azad University, Tabriz, Iran; *; 2 *Department of Basic Sciences, Faculty of Veterinary Medicine, Urmia University, Urmia, Iran.*

**Keywords:** Ciliary body, Histoanatomy, Lens, Ostrich

## Abstract

In the present study, the lenses and ciliary bodies of 20 ostrich eyes were studied macroscopically and microscopically. The histological slides were studied after staining by hematoxylin and eosin, Verhoeff, Van Gieson, and periodic acid-Schiff (PAS). Posterior surface of lens was more convex than its anterior surface. The average lens diameter and thickness were respectively measured as 1.43 ± 0.00 and 0.85 ± 0.00 cm. The average ciliary body thickness was measured as 1.48 ± 0.01 cm. In addition, the ciliary body was seen annular with mean horizontal and vertical external diameters as 4.80 ± 0.07 and 4.36 ± 0.06 cm, respectively. The retina is extended on ciliary body in this bird. The number of ciliary body processes was about 120. The epithelium of lens was cuboidal and the lens capsule had intense positive PAS reaction. Also, the anterior surface of capsule was thicker than its posterior surface. The lens fibers in the central part were thicker than other parts. Elastic and collagen fibers were not observed in the lens. The epithelium of ciliary processes had two layers; superficial cuboidal non-pigmented layer, and deep heavily pigmented layer. The ciliary body was supported by a hyaline cartilage. In addition to the smooth muscle fibers, many isolated skeletal muscle fibers were also seen in ciliary body. In conclusion, the lens and ciliary body of ostrich were similar to other birds, although there were little differences in anatomical dimensions and histological characteristics.

## Introduction

Eye is one of the important sensory organs of the body which plays a very important role in communications between the living creatures and their environment. Because of its importance and critical nature, many researchers have focused their studies on this issue.^1^^-^^3^ Some of the researchers have also studied the characteristics of the microscopic structure of the ostrich’s eye.^4^^-^^6^ Crampton in 1813 was the first to describe the ciliary muscle in ostrich and called it corneal depressor muscle. Afterward, in 1856, the description of similar structures in the eyes of birds was made by Brucke, who described the posterior localization of the choroid tensor muscle in owl and cassowary. Also in 1856, Muller studied the falcon eye and described presence of a posterior muscle that he divided as anterior (Muller's muscle) and posterior (Brucke's muscle) groups. These muscular groups represent different functions since they are anatomically separated.^7^

In modern anatomical classification, the avian ciliary muscle is formed by the Crampton's, Muller's, and Bruck's muscles. The avian ciliary muscle is different from that of mammals because it is striated and structurally similar to the skeletal muscle.^1^ The function of the avian ciliary muscle is still a source of debate. Some researchers suggest it controls the diameter of the Schlemn's canal and the angle of the sclera cleft, facilitating the aqueous humor draining.^5^ The role of the ciliary muscle in accommodation of the lens and cornea was demonstrated in the avian eye (chicken and pigeon).^8^ Accordingly, it was found that contraction of the ciliary muscle causes a change in the curvature of the cornea for corneal accommodation.^8^ The posterior ciliary muscle pulls the posterior ciliary body forward against the tension of the tenacular ligament. It was established that the lens plays a major role in the vertebrate accommodation; its optical properties during accommodation have been difficult to assess partly because the lens was located within the eye.^8^

It has been described that the ciliary muscle is divided by many muscular groups: one external group composed of the Crampton's muscle, which was connected to the tissue that covers the scleral ring bone; second group composed of the Brucke's muscle coupled to the sclera; and third group composed of the Muller's muscle that appears as a small segment of the Brucke's muscle.^9^ In contrast, the muscles involved in the eye accommodation have been studied previously and it has been suggested that the ciliary muscle is composed of only two muscular groups; distal and proximal, have been represented as the Crampton's and the Brucke's muscles, respectively.^10^

This work was proposed for determining the structure of the ciliary body and lens in eyes of the ostrich.

## Materials and Methods

In the present study, a total number of 20 eyes of African black adult ostriches were studied. First, the parts were anatomically studied for their appearance, dimensions, location and structure. Moreover, the dimensions were measured by ruler, calipers and eyepiece micrometer. For identifying their microscopic structure, lens and the ciliary body of the samples were cut and fixed in 10% formalin for at least 48 hr. In order to complete the fixation of the internal parts of the eye, it was necessary to inject 10% formalin into the eyeball. Seven days later, tissues were dried using autotechnicon apparatus (Hisure, Zhejiang, China) by passage of tissues through 70% to 100% ethanol and xylol to clear the tissues, and then paraffin blocks of the tissues were prepared. The specimens were cut in 5 μm slices and stained by hematoxylin and eosin (H & E; for the general study of the tissue), Verhoeff (for the elastic fibers), Van Gieson (for collagen fibers) and periodic acid-Schiff (PAS; for the existence of carbohydrates and glycogen). All histological sections were studied with a light microscope (Nikon Inc., New York, USA).^11^

## Results

**Fig. 1:**
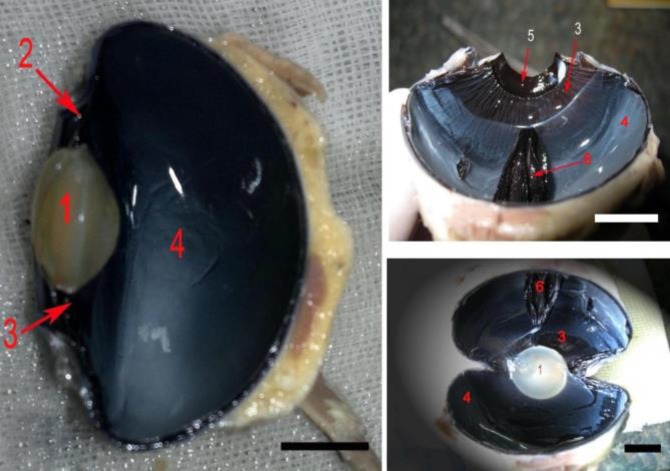
Anatomical sections of the ostrich eye. 1: Lens, 2: Ciliary body, 3: Ciliary processes, 4: Retina, 5: Iris, 6: Pectin

The eye lens of the ostrich was less convex in the anterior surface than the posterior one. In other words, the lens was less strongly curved in its corneal surface than vitreal surface (Fig. 1). In the ostrich, lenses were crystal, transparent and rather soft. They were surrounded and supported by the ciliary body (Fig. 1). The average diameter of the lens was 1.43 ± 0.00 cm, and its average thickness (anterior-posterior) was 0.85 ± 0.00 cm.

Some areas of the ciliary body which were the part of choroid layer were covered with retina and observed as completely round and radially arranged folds inside of the eye ball. The retina in this animal was passed the ciliary body and reached up to the contact point of the ciliary body and the lens (Fig .1). The average width of ciliary body was measured as 1.48 ± 0.01 cm. Moreover, the ciliary body was round and its mean outer horizontal and vertical diameters were 4.80 ± 0.07 and 4.36 ± 0.06 cm, respectively. The average number of ciliary processes in the ostrich was almost 120.

**Fig. 2:**
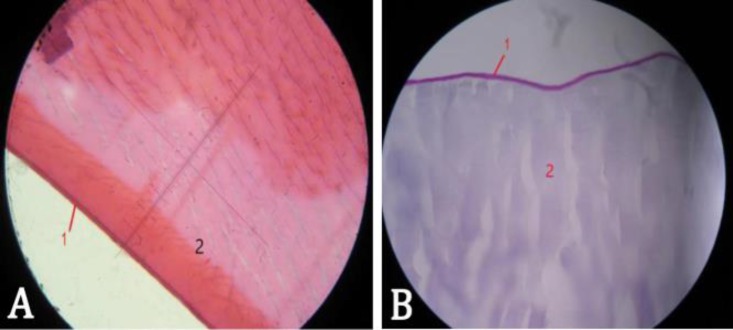
A) Histological section of lens (H & E, 100×), B) Histological section of lens (PAS, 100×). 1: Capsule 2: Lens fibers

The lens surface was surrounded by a thin basophilic capsule. The anterior epithelium was observed under the capsule, with one layer of squamous cells. These cells had almost round nuclei surrounding with light cytoplasm. The lens fibers were seen parallel to each other with an acidophilic color (Fig. 2). Some of the fibers had dark color while others were light-colored. The epithelium was thinner at the posterior part of the lens. The lens fibers were seemed to be thick in the middle areas, but they were looked thin in the lateral parts. The elastic fibers were not observed in the lens following Verhoeff staining. The lens capsule showed positive PAS reaction, while the lens fibers didn't show this kind of reactions (Fig. 2). In Van Gieson staining, the accumulation of collagen fibers was not observed in the lens. The lens capsule was 32 µm thick anteriorly and 5 µm posteriorly.

**Fig. 3:**
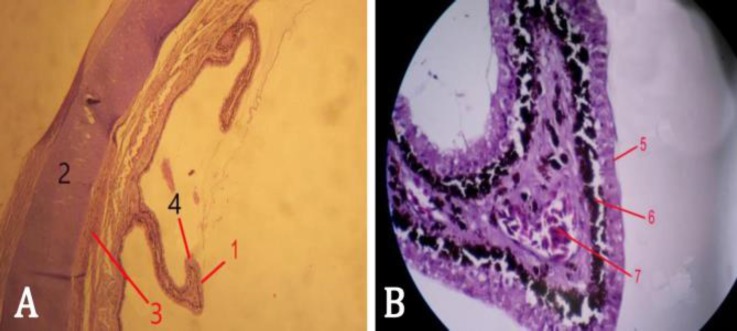
A) Histological section of ciliary body (H & E, 100×). B) Histological section of ciliary process (H & E, 400×). 1: Ciliary process, 2: Hyaline cartilage, 3: Skeletal muscle, 4: Double layer of epithelium, 5: Non-pigmented epithelium, 6: Pigmented epithelium, 7: Nucleated erythrocytes

The ciliary body and the processes had numerous lined wrinkles and folds which were shorter at the back. The connective folds were covered by pigmented epithelium. This epithelium was double-layered on the folds (Fig. 3). The superficial layer was non-pigmented cuboidal. The cells were shorter at the top of the folds, but they were long at the bottom. Some of the cells looked like pear or pot, and the neighboring cells seemed to be separated from each other. The deep layer was composed of melanin-containing cells having nuclei mostly covered by melanin.

Blood vessels with many red elliptical and nucleated erythrocytes were observed inside the folds (Fig. 3). The ciliary body as a part of the choroid layer was supported by a hyaline cartilage (Fig. 3). The main mass of the ciliary body was consisted of longitudinal smooth ciliary muscles as well as cross-sections of lymphatic and blood vessels. Skeletal muscular fibers were also seen in the form of separate masses in the ciliary body extended towards the ciliary process and the cornea (Fig. 3).

**Fig. 4:**
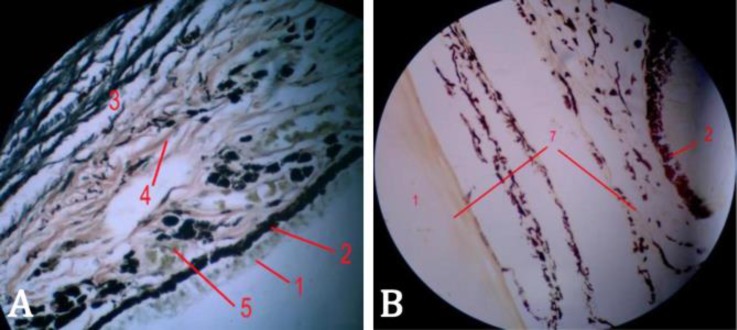
A) Histological section of the ciliary process (Verhoeff, 400×), B) Histological section of the ciliary body (Van Geison, 400×). 1: Non-pigmented epithelium, 2: Pigmented epithelium, 3: Elastic fibers, 4: Smooth muscles, 5: Nucleated erythrocytes, 6: Hyaline cartilage, 7: Collagen fibers

Periodic acid-Schiff reactions were only observed focally in superficial parts of some non-pigmented epithelial cells of the ciliary body. Moreover, blood vessel walls showed positive PAS reactions. The collagen fibers were not seen clear enough, but they were clearly observed in perichondrium and cartilage (Fig. 4). The elastic fibers inside the connective tissue of the ciliary body were observed in dark colors towards the ciliary process (Fig. 4).

## Discussion

The results of the present study showed that the convexity of ostrich lens in its anterior surface is greater than the posterior one. In other words, the ostrich eye lens is rather extended backwards in the posterior part. The same state was also observed in the eye lens of other animals.^13^ In some animals including rodents and marine mammals, the lens was reported to be round.^14^ The ciliary muscles in these animals are either weak or non-existent; therefore, they do not have the capacity to accommodation.^14^ The ostrich eye lens is rather soft, while it is much softer and more flexible in flying birds, helping quick reformation.^15^

On average, the diameter of the ostrich lens was 1.43 ± 0.00 cm, and its mean anterior-posterior thickness was 0.85 ± 0.00 cm. The chicken lens diameter and thickness have been reported 0.65 and 0.40 cm, respectively.^13^ In buffalos, the lens diameter has been measured as 1.80 ± 0.01 cm, and its thickness has been reported as 1.32 ± 0.02 cm.^3^ The bovine lens diameter may be a little less than its thickness. In other words, in cows, lens thickness has been proved to be more than lens diameter. The bovine lens thickness has been reported as 1.70 cm.^13^ The comparison of lens thickness and diameter between ostrich, ruminants, and chicken showed that diameter and thickness of lens in ostrich are less than that of ruminants and more than that of the chicken. It has been reported that the canine lens diameter is 1.00 cm, and its thickness is almost 0.70 cm.^16^

The ciliary body in the ostrich, which is the continuation of the choroid layer, in fact; is observed to be completely round and radially arranged folds inside the eye ball. In other words, some parts of the ciliary body are covered by retina. In other animals, the frontal limit of the retina is the starting point of the ciliary body, and it doesn't go beyond this limit.^16^ The mean width of the ciliary body in the ostrich was measured as 1.48 ± 0.01 cm. The mean width of the ciliary body in buffalo is 0.70 cm and the mean outer diameter of the ciliary body in buffalo is measured as 3.35 ± 0.70 cm.^3^ The number of ciliary processes in the ostrich is almost 120. In other animals, the number of such processes depends on the species and includes 70 to 100 ciliary processes. For example, 75 to 76 ciliary processes have been reported in the dog.^16^^,^^17^ The ciliary body and its processes provide a base on which lenticular zonules are attached. These zonules attach to the outer portions of the lens and hold it in place. The mentioned folds are connected to the lens by their fibers. The equinezonules are short and delicate at the beginning and become thick at the end.^18^ The thickness of the bovine ciliary body is variable, so, it has the greatest thickness in the posterior and lateral parts. The thickness of the ciliary body in cows ranges from 5.00 to 8.00 mm.^13^ The muscles of the ovine ciliary body have been reported to be very weak.^11^ In dogs, the ciliary processes have different lengths ranging from 0.10 to 2.40 mm.^18^ The width of the ciliary body and the number of ciliary processes in the ostrich were increased compared to other animals.

The lens fibers of the ostrich’s eye are dense and parallel to each other, and the surface of the lens is covered with a thick basophilic capsule. Lens capsule in the ostrich is 32 µm thick anteriorly and 5 µm posteriorly. In the adult canine lens, the capsule is 12.00 to 15.00 µm thick at the equator, 50.00 to 70.00 µm anteriorly, and only 2.00 to 4.00 µm thick posteriorly.^17^ Squamous epithelial cells, with almost round nuclei, cover the surface of the lens in the eyes of the ostrich. It was also found that the capsule is thicker in the anterior surface than the posterior part; this state was observed in other animals as well.^11^ The anterior surface of the lens capsule was observed to be red with extremely positive PAS reaction. Other animals have also reported to have elastic capsules with positive PAS reaction.^18^

Moreover, the lens fibers under the capsule are packed together, and the density of the fibers gets less, and the screen becomes clearer in the center. In other animals, on the lateral part of lens, the epithelial cells become longer, and form the fibers in the body of the lens after differentiation.^17^ Following Verhoeff staining, the elastic fibers were not observed in the ostrich lenses. The eye lens of buffalo has one central nucleus and two thick surrounding layers. In buffalo, lens does not have collagenous structure, and there are no signs of the elastic fibers existence.^3^ The eye lens of the ostrich, like most of animals, is made of three parts including the capsule, the epithelium, and the lens fibers. The eye lens is completely surrounded by the capsule including a sheet or several layers of collagen fibrils which have replaced the basal membrane materials.^12^ This state was also observed in the ostrich. Moreover, under the anterior capsule, the epithelium of the lens is located; consisting of a layer of cuboidal cells that the bottom of which is located towards the lens capsule, and their top is positioned towards the lens fibers. In most of animals, lens fibers are long cells extending from the posterior pole to its anterior pole, and the growth of the lens during life becomes feasible through continuation of differentiation and increase in the number of lens fibers. While the fibers are lengthened, the nucleus keeps its central position and comes out through the surface of the lens.^17^ Completely differentiated fibers do not have nucleus;^17^ this state exists in the ostrich as well. The mentioned fibers do not have organelles, and small particles are observed in their cyto-plasm following electron microscopy.^3^ In non-mammals animals, the lens has cylindrical radial fibers which help the lens to change its shape for accommodation.^14^

The ciliary body and processes have consecutive folds that become shorter backwards. The epithelium on the folds has two layers, and the superficial layer has cuboidal non-pigmented cells. The cells are shorter at the top of the folds and they are long at the bottom. In most of animals, the ciliary body is composed of two layers of cuboidal epithelial cells which are connected to each other on the top in a way that their basal membrane is located out-wards.^11^ Moreover, each ciliary process possesses a central core of stroma and blood vessels covered by a double layered epithelium.^18^ Other reports have indicated that epithelium of the ciliary process includes one outer pigmented and one inner non-pigmented layer.^19^ The epithelium of the two layers is cuboidal in most animals, and columnar in horses.^17^ In the ostrich, some cells were in the form of pear, and the neighboring cells were seemed to be separated in some parts. The connective tissue in the ciliary processes included smooth muscles with longitudinal cross-sections. Some cross-sections of the lymphatic and blood vessels in the form of arteries and veins were also observed. Moreover, the skeletal muscular fibers in the ciliary body of the ostrich were observed in the form of separate masses moving towards the ciliary and corneal processes. The smooth muscles in the ciliary body of carnivores are more developed than that of herbivores.^17^^,^^18^ In pigs, the anterior parts of the ciliary body muscles are transparent.^17^ In addition, the muscles in the ciliary body of carnivores are positioned horizontally at the equator in two internal and external surfaces.^16^ The ciliary muscle in domestic animals shows little growth and includes smooth muscular fibers with different thicknesses.^16^ In non-mammals animals including birds, the muscles of the ciliary body are observed in the form of skeletal muscles. In birds, at least two separate groups of skeletal muscles are observed in the ciliary body.^20^

At least two distinct bundles of muscle are positioned in this region of the avian eye, an anterior bundle arises near the margin of the cornea, and a posterior bundle, which is well developed in raptors such as the eagle and the hawk.^21^ Another report has indicated that muscles of the ciliary body in birds include anterior, posterior, and interior groups.^22^ The elastic fibers in the ciliary body of the ostrich were observed in dark color inside the connective tissue towards the ciliary process. In the other animals, thick elastic fibers with relatively greater frequency are also observed inside the connective tissue of the ciliary body.^17^

Generally, it can be concluded that apart from the differences in dimensions and minor differences in terms of microscopy, the lens and the ciliary body in the ostrich are similar to other birds and different from mammals.
